# Aging shapes baseline immunity in sterile-housed female hAPOE mouse genotypes

**DOI:** 10.46439/immunol.4.038

**Published:** 2025

**Authors:** F Fernandez, E Reyes-Reyes, D Chinnasamy, M Trial, KE Rodgers

**Affiliations:** 1 Center for Innovation in Brain Science, University of Arizona, Tucson, AZ, USA; 2 Department of Clinical and Translational Sciences, University of Arizona, Tucson, AZ, USA; 3 Department of Pharmacology, College of Medicine, University of Arizona, Tucson, AZ, USA

**Keywords:** Alzheimer’s disease, Apolipoprotein, Aging, Peripheral immunity, Housing and food sterility

## Abstract

The Apolipoprotein E ε4 allele (APOE4) is a major risk factor in the development of late-onset Alzheimer’s Disease (LOAD; AD) and has been associated with altered immunological responses, particularly under inflammatory challenge. Whether APOE genotype shapes baseline peripheral immunity across aging remains unclear. Because experimental context can influence immune phenotypes, we focus here on baseline profiles under specific-pathogen-free barrier housing (sterile housing) and discuss their implications. To this end, we highlight the peripheral immune profile in female humanized APOE mice (APOE3/3, APOE3/4, APOE4/4) maintained under sterile housing at 6, 9, and 15 months of age. Immunophenotyping of blood and spleen revealed significant age-related changes in B and T cell subpopulations and cytokine levels. Significant increases in activated and effector CD4^+^ and CD8^+^ T cells, as well as plasma cells, were observed at 15 months of age, particularly in the spleen. These shifts were primarily driven by ageing rather than APOE genotype. The only genotype-related differences detected were an increase in plasma TNF-α and IL-1β levels at 15 months and 9 months, respectively, in APOE4 compared with APOE3. Overall, aging exerts a stronger influence than APOE genotype on baseline peripheral immunity in female hAPOE mice under sterile housing, establishing an age-stratified baseline and providing a context-dependent rationale for future challenge-based studies to define genotype-by-inflammation interactions relevant to LOAD.

## Introduction

Alzheimer’s disease (AD) is a complex neurodegenerative disease commonly associated with the accumulation of β-amyloid (Aβ) plaques and neurofibrillary tangles in the brain [1]. Incidence and risk of AD development markedly increase at the age of 65, while continuing to exponentially increase every five years [2]. Contributing factors may include an increase in peripheral proinflammatory responses, Apolipoprotein E (APOE) genotypes, and endocrinological sex differences. The global frequency of the APOE4 allele is estimated to be 13.7% and is a significant risk factor in the development of late-onset Alzheimer’s Disease (LOAD) [3]. APOE4 homozygosity accounts for up to a 60% lifetime risk of AD by age 85 [4]. Chronic low-grade inflammation associated with immunosenescence (“inflammaging”) fosters peripheral immune dysregulation that can propagate neuroinflammation [5–7]. Importantly, these risk factors are particularly relevant to women, who are disproportionately affected by AD [8]. Together, these factors intersect across multiple mechanisms to drive AD susceptibility and progression.

Murine models remain essential for translational AD research, with lines varying in underlying pathophysiology, disease tempo, and severity [9]. To model aspects of LOAD risk, humanized APOE (hAPOE) targeted-replacement mice are widely used. Functional differences between APOE isoforms extend beyond lipid biology to immunity; notably, hAPOE4 has been associated with exaggerated pro-inflammatory responses under immune challenge compared with hAPOE3 [10]. Together with APOE4, ageing drives systemic biological changes, including cellular senescence and inflammaging, which can play critical roles in transitioning from immune quiescence to pro-inflammatory dysregulation [11,12]. Use of hAPOE4 mice has provided mechanistic insights into APOE-linked immune and metabolic pathways and has advanced our understanding of the underlying mechanisms contributing to LOAD.

Experimental context strongly shapes immune readouts. Husbandry and pathogen exposure—especially sterile housing—modulate immune state and disease modeling. The impact of the challenge-free conditions typical of sterile housing on immune responses in hAPOE mice remains unclear. Building on our prior work showing housing-dependent immune profiles in APOE4 mice [13], we examine baseline, age-stratified peripheral immunity across APOE genotypes under sterile housing, without deliberate stimulation. This baseline provides the foundation for challenge-based studies to test genotype–inflammation interactions relevant to LOAD.

## Methods in Brief

### Animals

Animals were housed in sterile conditions previously described [13]. Animal studies and procedures were conducted using the National Institutes of Health guidelines for procedures on laboratory animals and were approved by the University of Arizona Institutional Care and Use Committee. hAPOE3/3, hAPOE3/4, and hAPOE4/4 mice, originally from Jackson Laboratories, were bred and aged in a status A (helicobacter and murine norovirus negative, sterile food, and sterile housing) housing until 3 weeks before necropsy. At 6, 9, or 15 months, mice were culled and blood collected by cardiac puncture along with spleen. Tissues were processed as described in [13].

### Flow cytometry and cytokine measurement

White blood cells (WBC) and splenocytes were stained with antibodies and analyzed as described in [13]. Cytokine levels in plasma were measured using Meso Scale Discovery V-Plex Proinflammatory Panel 1 Mouse Kit (Cat #K15048D) according to the manufacturer’s instructions.

### Statistical analysis

Data was analyzed and prepared using GraphPad Prism 10 software. For flow cytometry and cytokine analysis, a standard two-way Analysis of Variance (ANOVA) with Post Hoc Tukey’s multiple comparisons test was performed. Data are represented as mean ± standard deviation. Statistical significance was defined as p<0.05.

## Study Overview

Female humanized APOE mice (APOE3/3, APOE3/4, and APOE4/4) maintained under sterile housing were assessed at 6, 9, and 15 months. Peripheral immune profiling of WBC and spleen was performed by multicolor flow cytometry focused on T cell and B cell populations, and plasma cytokines were quantified by immunoassays.

## Findings

### Age-related changes in T cell subsets are independent of APOE genotype in sterile-housed female hAPOE mice

**Figure 1.**
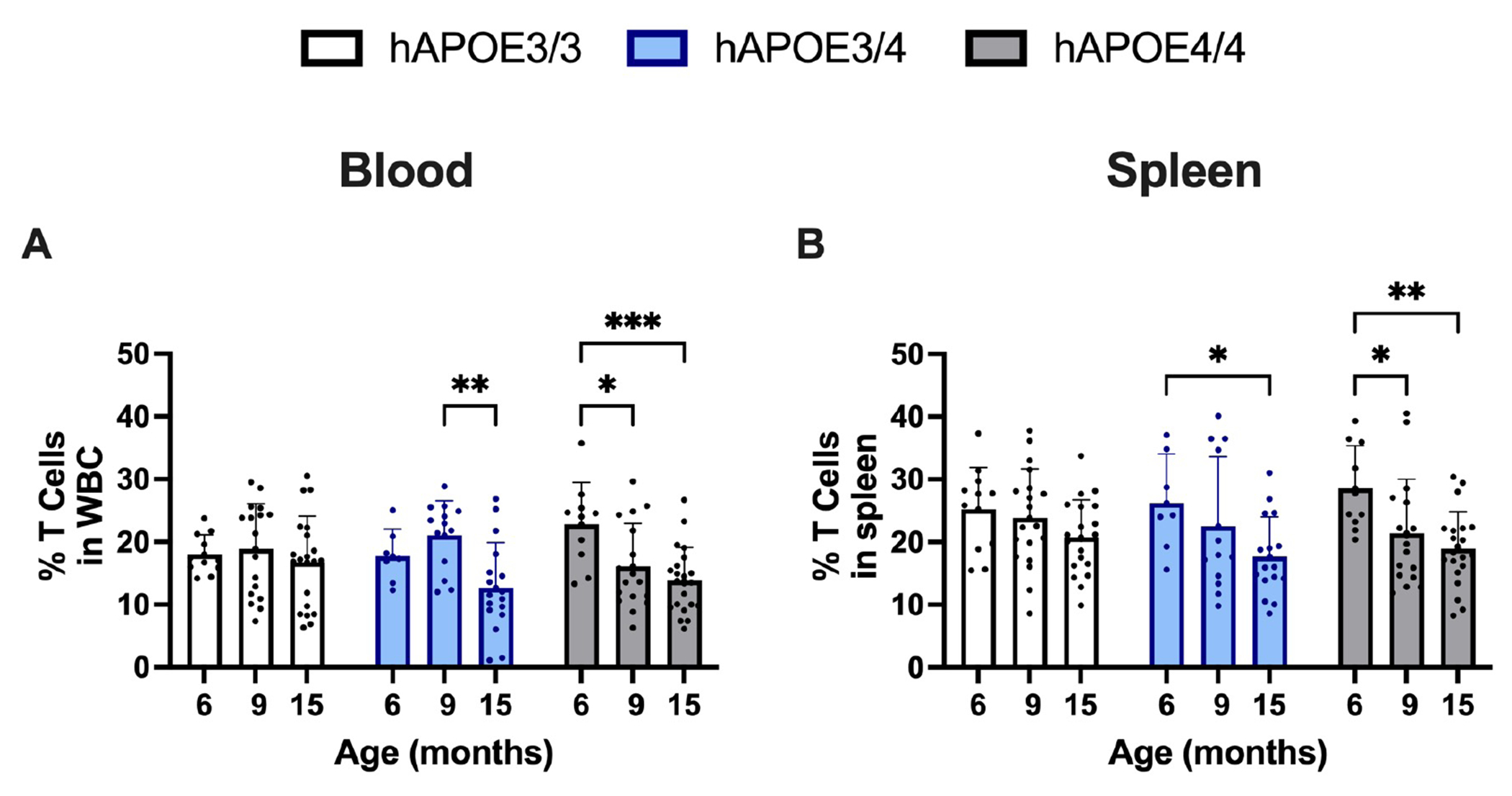
Percentage of T cells in both the spleen and blood at 15-months of age. Minor decreases in T cells were observed in the blood and spleen at 15-month of age across APOE4 carriers. *p≤0.05, **p≤0.01, ***p≤0.001, ****p≤0.0001.

Changes in T cell subset populations have been reported in APOE4 carriers, further highlighting the importance of T cells and their role in AD pathology [14]. Total T cells were unchanged across age in APOE3/3 but decreased in APOE 3/4 and APOE4/4 female mice at the age of 15 months compared to 6 months and 9 months (Figures 1A and 1B).

**Figure 2.**
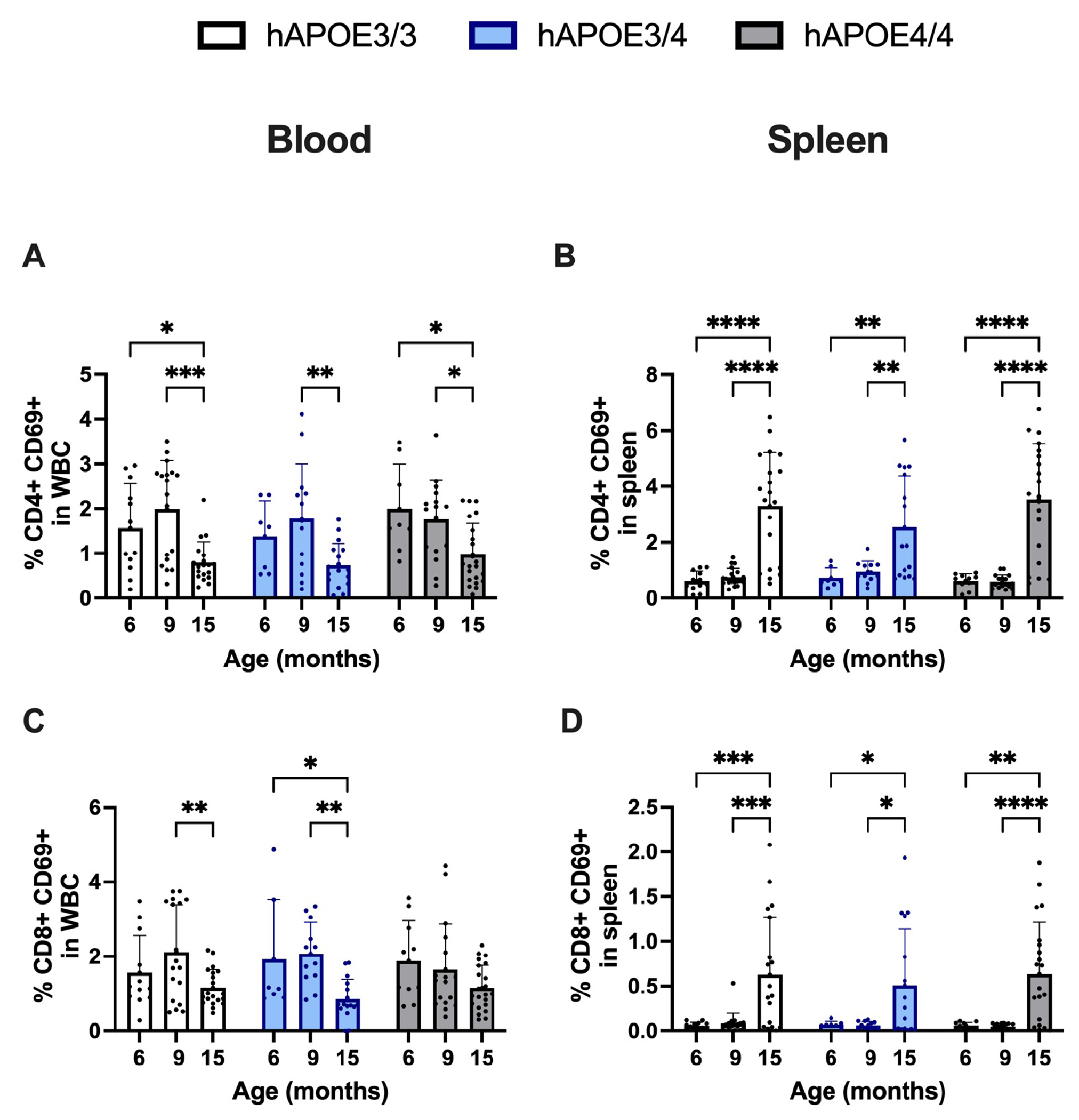
Percentage of activated (CD69 positive) CD4/CD8 T cell subset populations in the blood and spleen of female hAPOE mice. Significant increases in activated CD4/CD8 T cell subsets were observed at 15-months of age in the spleen. *p≤0.05, **p≤0.01, ***p≤0.001, ****p≤0.0001.

CD69 identifies early T cell activation and can also reflect tissue retention; although it is important to note that CD69 is not specific to naïve, memory, or effector subsets [15,16]. Activated CD4^+^ T cells in blood were reduced at 15 months versus 9 months across genotypes, whereas in spleen, they were higher at 15 months versus 6 months and 9 months in all genotypes (Figures 2A and 2B). Activated CD8^+^ T cells in blood also appeared to follow similar trends, with decreases observed at 15 months compared to 9 months in APOE3/3 and APOE3/4. However, no statistically significant changes were observed in APOE4/4 (Figure 2C). Notably, activated CD8^+^ T cells were increased in the spleen at 15 months versus 6 months and 9 months across all genotypes. Within each age group, values did not differ significantly among APOE3/3, APOE3/4, and APOE4/4 (Figure 2D).

**Figure 3.**
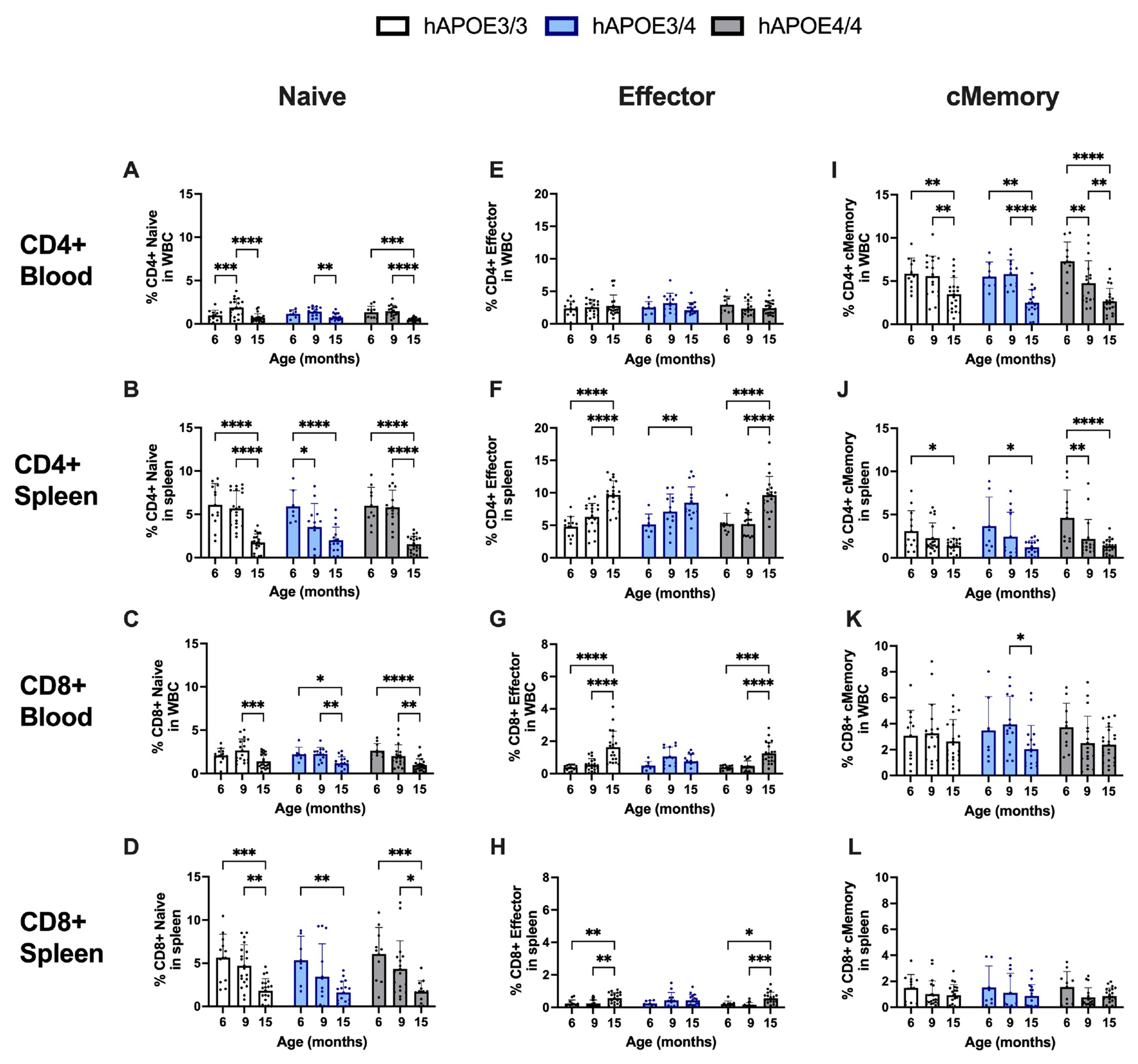
Percentage of T cell subsets, including CD4/CD8, naïve (**A–D**), effector (**E–H**) and central memory (**I–L**) T cell populations in the spleen and blood of female hAPOE mice. CD4 central memory and CD4/CD8 naïve T cell subsets decreased in both the spleen and blood at 15-months of age. Increases in spleen CD4 effector and spleen and blood CD8 effector populations were also observed. *p≤0.05, **p≤0.01, ***p≤0.001, ****p≤0.0001.

CD4^+^ and CD8^+^ naïve and CD4^+^ central memory populations were significantly decreased at 15 months of age compared to 6 and 9 months in both the blood and spleen, irrespective of genotype (Figures 3A–3D and 3I, 3J). On the other hand, CD8^+^ central memory populations stayed relatively consistent. Reductions in naïve and memory populations are a hallmark of immunosenescence, consistent with thymic involution over time. The differences in CD4^+^ and CD8^+^ central memory populations could be due to heterogeneity within these populations as a result of aging [17–19]. Percentages of CD4^+^ effector populations were significantly increased in the spleen, but not the blood, in all strains at 15 months of age (Figures 3E and 3F). CD8^+^ effector populations increased in both the spleen and blood at 15 months of age compared to 6 months and 9 months in APOE3/3 and APOE4/4, but not APOE3/4, genotypes (Figures 3G and 3H). Effector populations play a significant role in adaptive immune responses and can elicit a potent proinflammatory response; therefore, the survival of these subpopulations is carefully regulated [20]. The observed increases in these populations at 15 months of age in female hAPOE mice support age-related proinflammatory dysfunction. These observed changes support peripheral immune dysregulation; a condition often associated with aging. Overall, these results suggest that sterile housing conditions can play an important role in the immune response of female hAPOE animals, as the age-related changes in immune populations appear to be conserved across APOE genotypes.

### Age-related changes in B cell subsets are also independent of APOE genotype in sterile-housed female hAPOE mice

**Figure 4.**
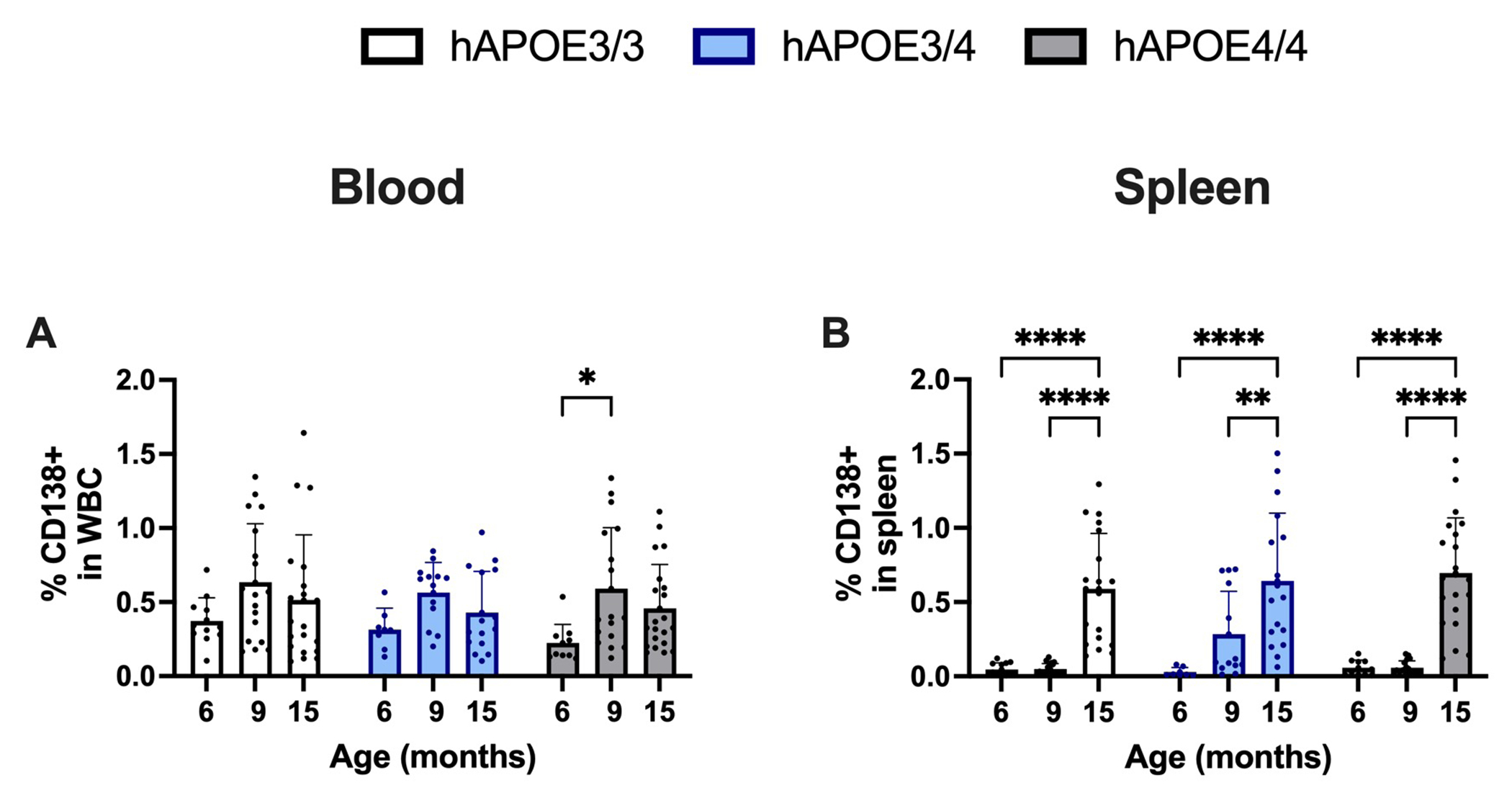
Percentage populations of plasma cells increase in the spleen (**B**) of sterile-housed female hAPOE mice at 15-months of age. Population changes are conserved across APOE genotype. *p≤0.05, **p≤0.01, ***p≤0.001, ****p≤0.0001.

B cells can play an important role in immune responses, contributing to AD pathology. B cells are responsible for antibody production and can modulate T cell differentiation, which can exacerbate proinflammatory responses [21]. Most B cell subpopulations remained consistent across all groups, with only an observed increase in splenic plasma cells (CD138^+^) noted at 15 months compared to 6 and 9 months of age. These changes occurred regardless of genotype (Figures 4A and 4B). Plasma B cells are terminally differentiated cells that are capable of antibody secretion, and increases in plasma cell populations are associated with autoimmune responses [22].

### Pro-inflammatory cytokine and chemokine levels in sterile-house female hAPOE mice

**Figure 5.**
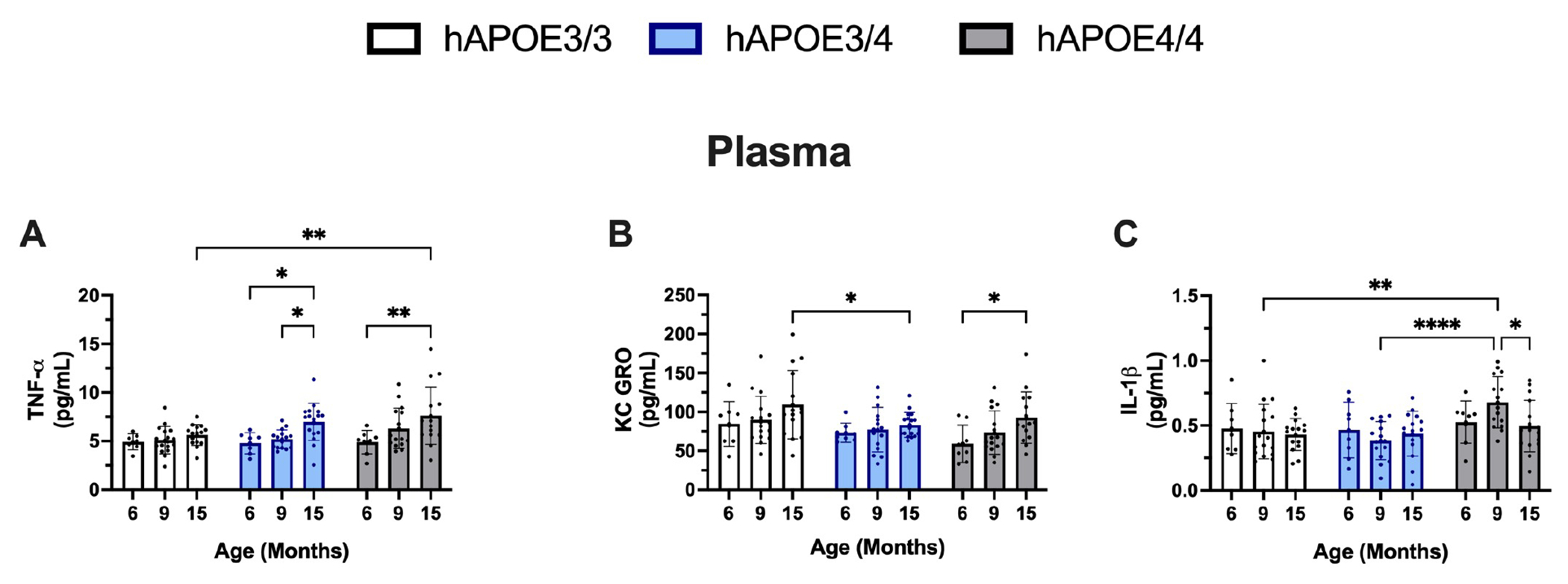
Plasma cytokine levels in sterile-house female hAPOE mice. TNF-α levels increase in APOE4 carriers at 15-months of age (**A**). KCGRO levels increase at 15-months in the APOE4/4 condition but not observed in other genotypes (**B**). IL-1β levels increase in the APOE4/4 genotype at 9-months of age (**C**). p≤0.05, **p≤0.01, ***p≤0.001, ****p≤0.0001.

Peripheral and central proinflammatory cytokines and chemokines are significant drivers of immune dysregulation in AD. Furthermore, proinflammatory cytokines and chemokines are also significantly higher in APOE4 carriers [23,24]. In contrast to the genotype-conserved cell frequencies at each age, changes in levels of two proinflammatory cytokines and one chemokine were observed in the plasma of sterile-housed female hAPOE mice. Levels of TNF-α in plasma significantly increased in APOE3/4 and APOE4/4 mice at 15 months of age compared to 6 and 9 months of age (Figure 5A). Levels of proinflammatory IL-1β in plasma were found to be the most elevated at 9 months of age in the APOE4/4 group. The observation of increased IL-1β in female APOE4/4 at 9 months of age may be of particular interest, as this age period correlates with estropause. The levels of Keratinocyte chemoattractant and human Growth-Regulated Oncogene (KCGRO), a proinflammatory chemokine, were also found to be elevated in the plasma of APOE4/4 mice at 15 months of age; however, the increase was not significant when compared to levels of KCGRO found in the APOE3/3 and APOE3/4 mice. These findings suggest that, in contrast to the T cell and B cell subset changes, which remained consistent across genotypes, changes in cytokines and chemokine levels displayed some level of genotype-specific responses.

## Significance

APOE4 carriers show distinct cerebrospinal proteomic profiles, irrespective of their AD state, with immune-pathway signatures associated with altered peripheral immune tone [25]. Such profiles may influence peripheral functionality, including antigen-processing/presentation pathways. APOE4 carriers have also been identified to have increased levels of activated T cells, potentially linked to heightened antigen presentation [26]. Together, these observations demonstrate the importance of the APOE4 polymorphism in modulating peripheral immune responses. However, in our baseline dataset under sterile housing, we did not detect genotype-associated differences in major leukocyte subsets. APOE4-associated differences are often most evident under stimulation (e.g., *in vivo* endotoxemia or systemic lipopolysaccharide (LPS) exposure), whereas baseline differences can be attenuated [27,28]. Sterile housing minimizes microbial experience, which can compress immune phenotypes and limit translatability to real-world physiology. Diverse microbial exposures (e.g., non-sterile, rewilded, or co-housed conditions) often produce more human-like, memory-skewed immune landscapes and can unmask environment-by-genotype interactions [13]. Although our study lacked a non-sterile arm, its controlled baseline is useful for interpreting when genotype differences should and should not be expected. Pathogen exposure shapes murine immunity [29]. Therefore, baseline findings from sterile housing should be contextualized when extrapolating to human settings and when designing future challenge studies. Consistent with this context, we view our null genotype finding at baseline as hypothesis-generating and potentially reflecting compressed immune variation under sterile housing, rather than the absence of genotype effects under stimulation. At baseline, immunophenotyping revealed that T and B cell populations were generally conserved across APOE genotypes, consistent with limited immune stimulation in sterile-housed mice. By contrast, we observed a significant age-related increase in splenic T and B cells with no detectable APOE genotype effect on these cell frequency endpoints. Changes in spleen immune cell populations have also been observed with ageing and can play a role in overall immune activity [30]. However, future studies should evaluate if these shifts are compatible with low-grade systemic inflammation (“inflammaging”) and altered trafficking/clearance of peripheral immune cells in sterilized-housing conditions.

Select proinflammatory cytokines showed genotype-associated elevations at specific ages (e.g., TNF-α at 15 months in APOE3/4 and APOE4/4; IL-1β at 9 months in APOE4/4), but these differences were not mirrored by between-genotype differences in leukocyte subset frequencies at the same ages. This partial disconnect likely reflects that soluble mediators and cell-frequency endpoints capture different aspects of immune state and may differ in sensitivity at baseline. Under sterile housing, minimal microbial exposure can compress immune phenotypes and may reduce the detectability of genotype effects in cross-sectional cell frequencies; however, without a non-sterile comparison, we cannot attribute this pattern to housing per se. The consistent age-related shifts across genotypes indicate that aging is the primary driver at baseline and motivate challenge-based experiments (or more microbially complex contexts) to test whether APOE-related differences emerge more prominently.

The identified age-related changes in both spleen and blood immune populations, accompanied by increases in cytokines, show relevance to the human AD state, as inflammatory responses have been identified as a central mechanism [31,32]. Similarly, dysregulation of CD4^+^ effector T cells— for example, has been demonstrated to play a role in exacerbating cognitive decline and promoting AD pathology in a transgenic mouse model [33]. Increases in plasma and cerebrospinal levels of TNF-α have also been identified to occur early in AD and are associated with increases in cognitive impairment [34]. Because AD is tightly linked to aging, our data supports the idea that age-related shifts in peripheral immunity may be disease-relevant; however, our results are observational and baseline-only. We therefore refrain from causal claims and emphasize that mechanistic studies (including challenge paradigms or microbially complex housing) are needed to determine whether and how these shifts influence onset or progression.

## Conclusion

In sterile housing at baseline, aging—not APOE genotype—drives peripheral immune remodeling in female hAPOE mice. Across genotypes, leukocyte frequencies did not differ within age strata, and only a few, age-specific APOE4-associated cytokine elevations were observed. Whether APOE effects become detectable likely depends on context: (1) apply standardized immune challenges to probe stimulation-dependent differences; (2) use housing conditions with greater, real-world microbial exposure to test environment–genotype interactions; and (3) include functional readouts, not only cell counts. Because our analysis focused on female mice at baseline in sterile housing, conclusions are intentionally cautious and non-causal. Future work should add male cohorts, examine microbially diverse settings, and use defined challenges to determine when and how APOE genotype shapes peripheral immunity in LOAD-relevant models.

## Supplementary Material

PB-2025-325-Supplementary-FIle
