# Supplementary material for: Aging shapes baseline immunity in sterile-housed female hAPOE mouse genotypes
Source: J Cell Mol Immunol. Author manuscript; Available in PMC 2026 Jul 29. (PMC13411058; doi:10.46439/immunol.4.038)
Supplement: PB-2025-325-Supplementary-FIle [file NIHMS2193210-supplement-PB-2025-325-Supplementary-FIle.pdf]

Citation: Fernandez F, Reyes-Reyes E, Chinnasamy D, Trial M, Rodgers KE. Aging shapes baseline immunity in sterile-housed female hAPOE mouse genotypes. J Cell Mol Immunol. 2025;4(1):33-39.

Supplementary Information

Table S1. Sample size of sterile-housed, female hAPOE mice used in the study.

**Group**	**6 Months**	**9 Months**	**15 Months**	**Total**
APOE3/3	12	18	20	50
APOE3/4	7	13	17	37
APOE4/4	10	17	19	46
**Total**	29	48	56	133

Table S2. List of antibodies used in differentiation panels for flow cytometry analysis.

**T cell differentiation**			
**Antibody**	**Fluorophore**	**Clone**	**Manufacturer**
CD45	VioGreen	REA737	Miltenyi
CD44	VioBright FITC	REA664	Miltenyi
CD62L	PE	REA828	Miltenyi
CD8b	PerCP-Vio700	REA793	Miltenyi
CD69	PE-Vio770	REA937	Miltenyi
CD3 epsilon	APC	REA606	Miltenyi
CD4	APC-Vio770	REA604	Miltenyi
**B cell differentiation**			
**Antibody**	**Fluorophore**	**Clone**	**Manufacturer**
CD45R (B220)	VioGreen	REA755	Miltenyi
IgD	FITC	REA772	Miltenyi
CD19	PE	REA749	Miltenyi
MHCII	PerCP-Vio700	REA805	Miltenyi
CD138	PE-Vio770	REA104	Miltenyi
CD3 epsilon	APC	REA606	Miltenyi
IgM	APC-Vio770	REA979	Miltenyi
